# The Iron-Sulphur Cluster Biosynthesis Regulator IscR Contributes to Iron Homeostasis and Resistance to Oxidants in *Pseudomonas aeruginosa*

**DOI:** 10.1371/journal.pone.0086763

**Published:** 2014-01-22

**Authors:** Adisak Romsang, Jintana Duang-Nkern, Panithi Leesukon, Kritsakorn Saninjuk, Paiboon Vattanaviboon, Skorn Mongkolsuk

**Affiliations:** 1 Department of Biotechnology, Faculty of Science, Mahidol University, Bangkok, Thailand; 2 Center for Emerging Bacterial Infections, Faculty of Science, Mahidol University, Bangkok, Thailand; 3 Molecular Medicine Graduate Program, Faculty of Science, Mahidol University, Bangkok, Thailand; 4 Laboratory of Biotechnology, Chulabhorn Research Institute, Bangkok, Thailand; 5 Program in Applied Biological Science: Environmental Health, Chulabhorn Graduate Institute, Bangkok, Thailand; 6 Center of Excellence on Environmental Health and Toxicology, Ministry of Education, Bangkok, Thailand; CINVESTAV-IPN, Mexico

## Abstract

IscR is a global transcription regulator responsible for governing various physiological processes during growth and stress responses. The IscR-mediated regulation of the *Pseudomonas aeruginosa isc* operon, which is involved in iron-sulphur cluster ([Fe-S]) biogenesis, was analysed. The expression of *iscR* was highly induced through the exposure of the bacteria to various oxidants, such as peroxides, redox-cycling drugs, intracellular iron-chelating agents, and high salts. Two putative type 1 IscR-binding sites were found around RNA polymerase recognition sites, in which IscR-promoter binding could preclude RNA polymerase from binding to the promoter and resulting in repression of the *isc* operon expression. An analysis of the phenotypes of mutants and cells with altered gene expression revealed the diverse physiological roles of this regulator. High-level IscR strongly inhibited anaerobic, but not aerobic, growth. *iscR* contributes significantly to the bacteria overall resistance to oxidative stress, as demonstrated through mutants with increased sensitivity to oxidants, such as peroxides and redox-cycling drugs. Moreover, the regulator also plays important roles in modulating intracellular iron homeostasis, potentially through sensing the levels of [Fe-S]. The increased expression of the *isc* operon in the mutant not only diverts iron away from the available pool but also reduces the total intracellular iron content, affecting many iron metabolism pathways leading to alterations in siderophores and haem levels. The diverse expression patterns and phenotypic changes of the mutant support the role of *P. aeruginosa* IscR as a global transcriptional regulator that senses [Fe-S] and directly represses or activates the transcription of genes affecting many physiological pathways.

## Introduction

Iron-sulphur clusters ([Fe-S]) are important components of many enzymes involved in diverse cellular processes, such as DNA repair, gene regulation, RNA modifications, biosynthetic pathways, aerobic and anaerobic respirations, and nitrogen and carbon metabolism [1]. Approximately 5% of the total proteins in *Escherichia coli* are [Fe-S]-containing proteins [1]. Three different types of bacterial [Fe-S] biosynthetic systems, Isc (*i*ron-*s*ulphur *c*luster), Suf (*su*lphur*f*ormation) and Nif (*ni*trogen *f*ixation), have been characterised [1], [2]. The Isc and Suf systems are involved in the maturation of most [Fe-S]-containing proteins in the cell, while Nif contributes to the maturation of nitrogenase in certain nitrogen-fixing bacteria [3], [4]. In many bacteria, the Isc machinery is encoded by the conserved *iscRSUA-hscBA-fdx* (*iscR*, iron-sulphur cluster assembly transcription factor; *iscS*, cysteine desulphurase; *iscU*, [Fe-S] assembly scaffold; *iscA*, [Fe-S] assembly protein; *hscB*, [Fe-S] protein assembly co-chaperone; *hscA*, [Fe-S] protein assembly chaperone; and *fdx*, ferredoxin) gene cluster in the *isc* operon in a housekeeping pathway for [Fe-S] biogenesis [5].

Iron is important for bacterial growth and survival. The iron concentration and availability are crucial; too much iron leads to severe oxidative stress, and too little iron severely hinders bacterial growth. Bacteria have evolved mechanisms to maintain a precise intracellular iron concentration, including the iron storage proteins (such as ferritins) and a general ferric iron buffering system (such as haem and [Fe-S] cluster) [6], [7]. IscR acts as a sensor of cellular [Fe-S] levels and a global transcription regulator for [Fe-S] biogenesis under both physiological and stressful conditions [8], [9]. In *Escherichia coli,* IscR exists in two major forms: apo-IscR lacks the [2Fe-2S] cluster and [2Fe-2S]-IscR has the [2Fe-2S] ligated to IscR. In addition, two IscR binding motifs, type 1 and type 2, have been characterised [10]. [2Fe-2S]-IscR binds to both types of binding motifs, whereas apo-IscR only binds to the type 2 motif [11]. In *E. coli*, IscR is a transcriptional repressor of the *isc* operon [12]. [2Fe-2S]-IscR binds to the type 1 IscR-binding motif (5′ATASYYGACTRWWWYAGTCRRSTAT3′) and represses the transcription of the *isc* operon [8], [13]. A proposed model was showing that the level of [2Fe-2S]-IscR is reduced during oxidative stress and iron limiting conditions, leading to derepression of the *isc* operon [12]. In *E. coli*, the post-transcriptional regulation of the *isc* gene expression is mediated through the Fur (ferric uptake regulator)-regulated small non-coding RNA RyhB [14]. Moreover, the expression of the *suf* operon (*sufABCDSE* gene cluster) is activated by IscR [9]. *suf* operon expression has been shown to be induced by stresses implying it plays a role in adaptation to stressful conditions [15].

*Pseudomonas aeruginosa* is a major opportunistic human pathogen that causes serious infections in hospitalised patients, particularly patients with cancer, cystic fibrosis, and burn injuries. The *P. aeruginosa* PAO1 genome contains a homologue of the *isc* operon [16] but lacks the putative *suf* operon [16], [17]. The *iscR* gene is required for H_2_O_2_ protection in *P. aeruginosa* PA14 [18]. The inactivation of *iscR* reduced KatA catalase activity and attenuated virulence in *Drosophila melanogaster* and mouse peritonitis models [19]. Nonetheless, the regulation of the *P. aeruginosa isc* operon has not been characterised. *P. aeruginosa* IscR has been implicated as a global regulator of many cellular responses, including oxidative stress [20]. In the present study, we showed that IscR regulated the *isc* operon for [Fe-S] biogenesis under both physiological and stress-induced conditions. In addition, *iscR* is also important in maintaining overall iron homeostasis and its inactivation leads to a phenotype that indicates iron limitation.

## Results and Discussion

**Figure 1:**
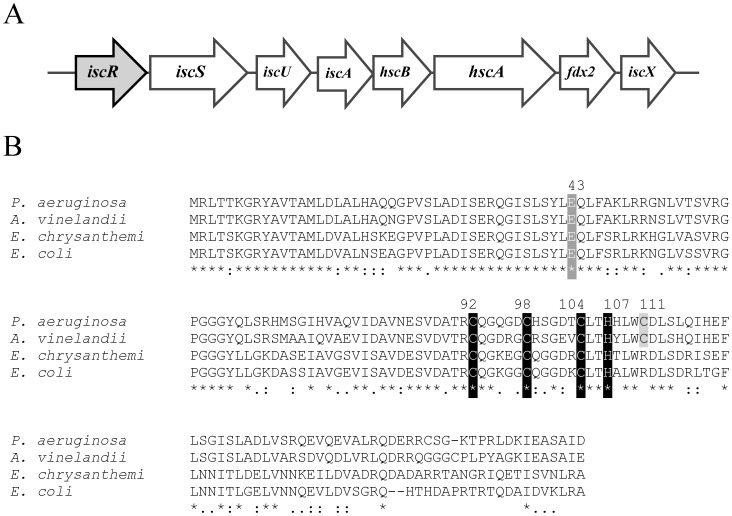
Gene organisation of *isc* gene cluster and multiple alignments of *P. aeruginosa* IscR. (A) Gene organisation of *isc* gene cluster in *P. aeruginosa* PAO1 [16]. (B) Alignments of IscR from *P. aeruginosa* with IscR sequences from other bacteria. The alignments were performed using the CLUSTALW algorithm. Black and grey boxes indicate the amino acids responsible for the iron-sulphur cluster ligation, and C111, respectively. The asterisk, colon, and period symbols indicate identical residues, conserved substitutions, and semi-conserved substitutions, respectively. The number on top of the alignments indicates the position of the indicated amino acid.

The analysis of the *P. aeruginosa* PAO1 genome sequence [16] revealed the homologues of the *isc* gene cluster *iscRSUA-hscBA-fdx2-iscX* (PA3815-PA3808) that could be involved in [Fe-S] biogenesis. The PAO1 *isc* operon gene organisation shares many similarities to that of the *E. coli isc* operon [8], [12]. The minor differences are the lack of *rhyB* binding site between *iscR* and *iscS* and additional *iscX* (Figure 1A). Northern blot analysis, probing with either the coding sequence of *iscR* or *fdx2*, showed a similar positively hybridised band of approximately 5 kb, suggesting that this gene cluster is transcribed as an operon (data not shown). No homologues of the *suf* operon were identified in the PAO1 genome. IscR regulates the *isc* operon in *E. coli* [12], [21]. *P. aeruginosa* IscR shares 78%, 65% and 67% identity with the IscR from *Azotobacter vinelandii*, *Erwinia chrysanthemi* and *E. coli*, respectively [12], [22], [23]. The residues involved in [Fe-S] ligation (C92, C98, C104, H107) [13] and E43, which prevents the apo-IscR from binding to the type 1 binding site [11], are all conserved (Figure 1B). Nonetheless, no putative *ryhB* or *P. aeruginosa* sRNA *prrF* (a *rhyB* analogue) binding motifs were identified between the *iscR* and *iscS* intergenic sequences.

### Stress-induced Expression Profile and Promoter Analysis of the *isc* Operon

**Figure 2:**
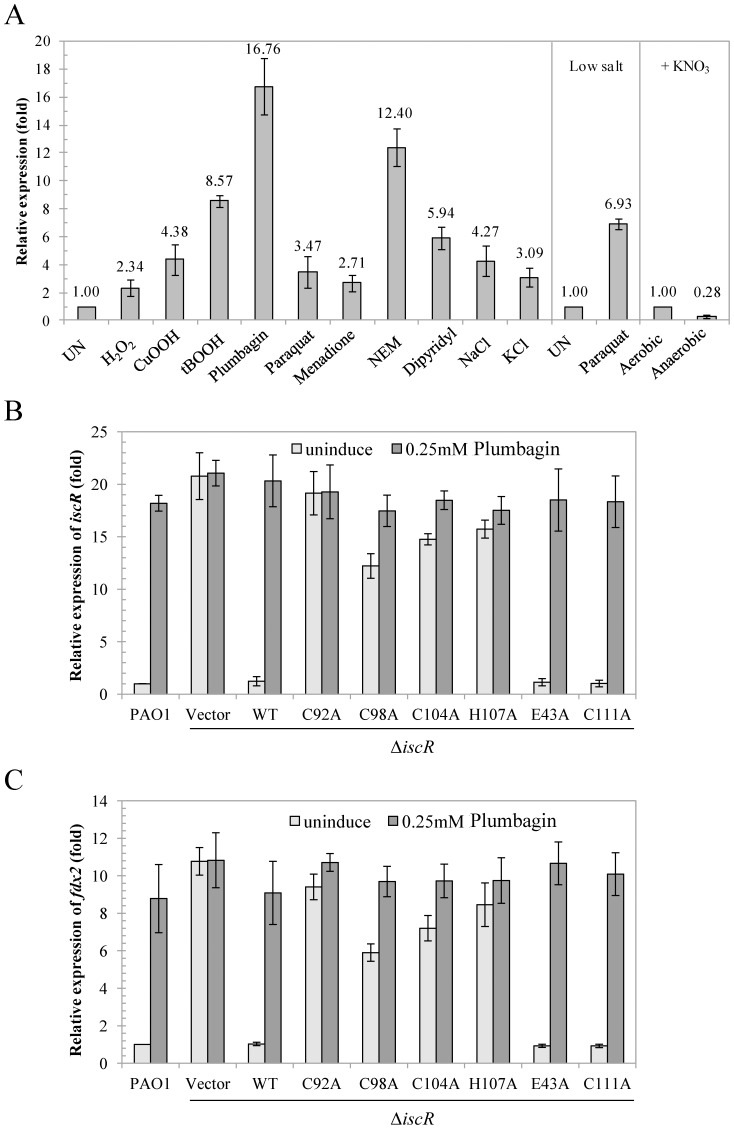
Expression levels of the *isc* operon in response to stresses. (A) Analysis of the *iscR* expression during normal growth and in response to oxidants after inducing the culture with 1 mM H_2_O_2_, 1 mM CuOOH, 1 mM tBOOH, 0.5 mM paraquat, 0.5 mM menadione, 0.5 mM plumbagin, 0.1 mM NEM, 1 mM dipyridyl, 0.5 M NaCl, 0.5 M KCl, LB medium without NaCl (low salt) without (UN) and 0.5 mM paraquat for 15 min under aerobic and anaerobic conditions supplemented with nitrate (+KNO_3_). (B) and (C) show the expression profiles of *iscR* and *fdx2*, respectively, in uninduced (grey bars) and 0.25 mM plumbagin-induced (closed bars) cultures of *P. aeruginosa* PAO1 and Δ*iscR* mutant harbouring pBBR1MCS-4 plasmid (vector), plasmid expressing wild-type IscR (WT), and mutated IscR (C92A, C98A, C104A, H107A, E43A, or C111A). qRT-PCR was performed as described in the Methods. The data are presented as the means ± SD from three independent experiments. The relative expression was analysed using the 16S rRNA gene as the normalising gene and expressed as fold expression relative to the level of the uninduced PAO1.

The lack of the *suf* operon in *P. aeruginosa* suggests that the *isc* operon functions in [Fe-S] biosynthesis under both housekeeping and stressful conditions. IscR is a global regulator involved in the regulation of genes from diverse physiological functions, hence the expression of IscR is likely important for the ability of *P. aeruginosa* to adapt to changing conditions. qRT-PCR was performed to determine the expression levels of the genes in the *isc* operon (*iscR* and *fdx2*) in response to an intracellular iron-chelating agent (1 mM 2,2′-dipyridyl) and various stresses, including hydrogen peroxide (1 mM H_2_O_2_), organic hydroperoxides (1 mM cumene hydroperoxide [CuOOH] or t-butyl hydroperoxide [tBOOH]), redox-cycling drugs (0.5 mM plumbagin, paraquat, or menadione), thiol-depleting agent (0.1 mM N-ethylmaleimide [NEM]), and high salt conditions (0.5 M NaCl or KCl). The results of the expression analysis illustrated that *iscR* expression was highly induced (4.4–16.8-fold) upon treatment with plumbagin, organic hydroperoxides, dipyridyl, and NEM and to a lesser extent (2.3–4.3-fold) through paraquat, menadione, H_2_O_2_ and high salt treatments (Figure 2A). The induction of *iscR* through paraquat is affected by the salt concentration in the media. A high salt concentration inhibits paraquat transportation into the cells; thus, lowering the salt concentration in the medium enhances the paraquat-mediated induction of *iscR* expression (Figure 2A). The expression of the *fdx2* (PA3809) gene was used to monitor gene expression in the *isc* operon. The *fdx2* expression profiles in response to plumbagin-induced stress were comparable to that of *iscR* (Figure 2B and C). These observations indicate that the expression of the *isc* operon is induced through iron depletion (dipyridyl treatment), high salts, thiol depletion and oxidative stresses (peroxide and redox-cycling agents). These conditions affect not only the iron availability but also damage [Fe-S], resulting in the overall reduced availability of [Fe-S]. Thus, increased [Fe-S] biogenesis and the up-regulation of the *isc* operon are required to maintain [Fe-S] homeostasis under stressful conditions.

**Figure 3:**
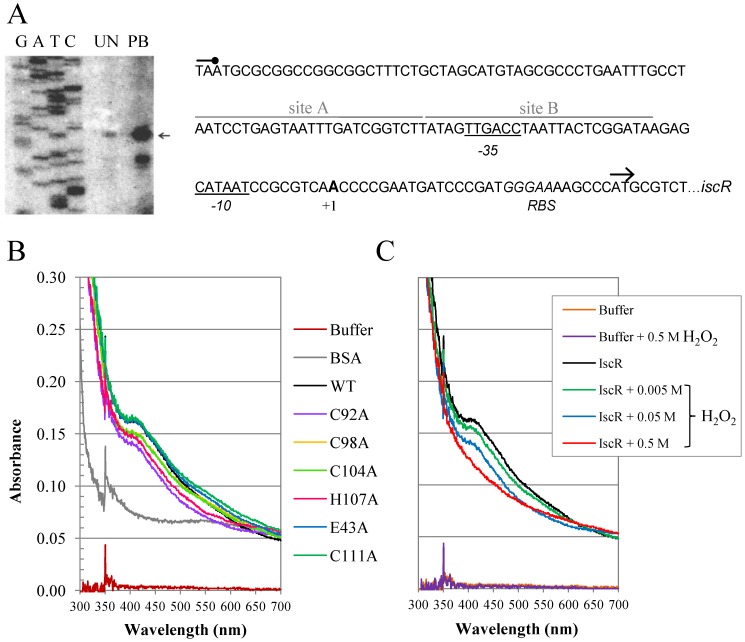
Characterisation of the *iscR* promoter. (A) The primer extension assay was performed using ^32^P-labelled primer BT3577 and RNA extracted from PAO1 grown under uninduced (UN) and 0.25 mM plumbagin-induced conditions. The extension products were separated on an 8% acrylamide-7 M urea sequencing gel. G, A, T, and C represent the DNA ladder sequence prepared using ^32^P-labelled primer BT3577 and putative *iscR* promoter fragments as templates. The arrowhead indicates the transcription start site (+1). The −10 and −35 elements are underlined. The consensus sequence of type 1 *E. coli* IscR binding site is aligned above the sequence line in corresponding letters, and the homologous bases are marked with asterisks. The putative ribosome-binding site (RBS) is indicated in italic type. (B) UV-visible absorption spectra of various IscR variants (C92A, C98A, C104A, H107A, E43A, or C111A). Purified IscR proteins in 50 mM phosphate buffer (10 µM) were used in the experiments. BSA (10 µM) was used as non-[Fe-S] protein control. (C) UV-visible absorption spectra of wild-type IscR treated with the indicated concentrations of H_2_O_2_.

Next, a primer extension experiment was performed to determine the transcriptional start site (+1) of the *isc* operon. The +1 was mapped to A residue located at 29 bases upstream of the ATG translation start codon of *iscR* (Figure 3A). The putative −35 and −10 promoter elements, separated by 18 bases, were identified as TTGACC and CATAAT, respectively. The results of the *iscR* primer extension showed several-fold more products in plumbagin-induced samples. Thus, these results confirmed the qRT-PCR analysis, implicating plumbagin as a potent inducer of *iscR* expression (Figure 2B). In addition, these results show that the stress induced up-regulation of *iscR* results from increased transcription initiation from the promoter.

Analysis of sequences upstream of the *iscR* transcription start revealed two putative type 1 IscR-binding motifs denoted site A (5′AATCCTGAGTAATTTGATCGGTCTT3′) and site B (5′ATAGTTGACCTAATTACTCGGATAA3′) located between positions −18 to −67 (Figure 3A). These two motifs had 68% and 76% identity, respectively, to the consensus sequence for the *E. coli* type 1 IscR-binding motif (5′ATASYYGACTRWWWYAGTCRRSTAT3′) [10]. This suggests autoregulation of *iscR*-expression in response to varying [Fe-S] demand. The location of these two type 1 sites overlaps with the RNA polymerase recognition motif (Figure 3A). Thus, the binding of IscR to these sites would be expected to prevent RNA polymerase binding to the promoter and inhibit the transcription of *isc* operon. A recent report indicates that *E. coli* has three IscR binding sites upstream of the *iscR* promoter that are involved in regulation of the *iscR* operon [8]. The possible role of the IscR binding sites within the *P. aeruginosa iscR* promoter region is being investigated.

### IscR is a Transcriptional Regulator of the *isc* Operon

The *iscR* mutant was constructed through gene deletion. The expression of *iscR* and *fdx*2 was used to monitor the expression of the *isc* operon in PAO1 and a Δ*iscR* mutant. qRT-PCR analysis of *iscR* was performed using forward primers EBI120 (located at position +1 to +18 of *iscR* transcript [Figure 3A], hence outside a ribosome binding site and ATG of the *IscR* and the gene deletion site of the Δ*iscR* mutant) and BT3613 (located in coding region of *iscR*). These primers facilitate the analysis of Δ*iscR* mutants carrying Tn7T*iscR*, pBBR*iscR* or various site-directed *iscR* mutations. The results showed that under uninduced conditions, the expression of *iscR* and *fdx2* in the Δ*iscR* mutant was 20.8- and 10.8-fold higher than the levels in PAO1, respectively. Plumbagin treatments did not further enhance the expression of either *iscR* or *fdx2* in the Δ*iscR* mutant (Figure 2B and C). The expression of *iscR* from a Tn7T vector in the mutant led to the repression of *iscR* and *fdx2* expression to levels similar to those observed in PAO1 (Figure 2B and C). Furthermore, the plumbagin-induced expression of both genes in the complemented strain was restored to wild-type levels (Figure 2B and C).

Additional experiments were performed to determine the form of IscR and type of binding site involved in the repression of the *isc* operon. Two major forms of the transcription regulator, apo-IscR and [2Fe-2S]-IscR have been characterised [13]. Apo-IscR binds to type 2-binding sites with greater affinity than type 1 sites. This is thought to be due to the inhibitory effect of residue E43 during apo-IscR binding to type 1 sites based on the observation that an E43A mutation enhances the binding of apo-IscR to type 1 sites in the absence of [2Fe-2S] ligation [11]. The residue E43 is conserved in *P. aeruginosa* IscR. Experiments were performed to test the effect of wild-type IscR and IscR-E43A on the regulation of *iscR* operon expression in the Δ*iscR* mutant. Our results showed that, similar to the wild-type IscR, IscR-E43A repressed *iscR* and *fdx2* expression in the Δ*iscR* mutant (Figure 2B and C). IscR-E43A repressed *iscR* and *fdx2* transcription to levels similar to those observed in a wild-type IscR background, this indicated that that E43 is not required for IscR-mediated repression of the *iscR* promoter.

The IscR residues, C92, C98, C104, and H107, which serve as ligands for [2Fe-2S] cluster ligation [13] are conserved in *P. aeruginosa* IscR. Experiments to determine whether [2Fe-2S]-IscR is involved in the regulation of the *isc* operon were performed through mutating C92, C98, C104, and H107 residues to alanine (A) residues in *P. aeruginosa* IscR. The plasmids containing the mutagenised *iscR*, i.e., pBBR*iscR*-C92A, pBBR*iscR*-C98A, pBBR*iscR-*C104A, and pBBR*iscR*-H107A, were transferred into the *iscR* mutant, and the restoration of plumbagin-induced *iscR* and *fdx2* expression was tested. The concentration of plumbagin was reduced to 0.25 mM due to the plumbagin hypersensitive phenotype of the Δ*iscR* mutant. The expression of *iscR* fully complemented the constitutive high and oxidant non-inducible expression of *iscR* and *fdx2* (Figure 2B and C). The Δ*iscR* mutant harbouring pBBR*iscR* repressed expression of *iscR* (16.9-fold) and *fdx2* (10.5-fold) compared with the *iscR* mutant harbouring empty vector. The plumbagin treatment of these cells induced the expression of both *iscR* and *fdx2* to the levels attained in PAO1 harbouring the vector control (Figure 2B and C). The expression of mutated C92A and H107A *iscR* did not repress the expression of these genes while the IscR-C98A and IscR-C104A mutants showed 1.6- and 1.4-fold repression of *iscR* expression compared with the 16.9-fold expression observed with wild-type IscR. Similar expression patterns were obtained for the *fdx2* analysis. Nonetheless, plumbagin treatment induced the expression of target genes to levels comparable to those obtained with wild-type IscR. The inability of mutant IscR-C92A and IscR-H107A to repress *iscR* and *fdx2* expression suggests that these mutations prevent the ligation of [2Fe-2S] to IscR, which is sufficient to prevent the repression of the *isc* operon. Surprisingly, IscR-C98A and IscR-C104A produced intermediate repression levels and plumbagin inducible expression patterns, implying that these IscR mutants might weaken the attachment of [2Fe-2S] to IscR. Under aerobic conditions, the weakened [2Fe-2S] attachment to mutated IscR that might render [2Fe-2S]-IscR more susceptible to aerobic oxidation, resulting in a mixture comprised primarily of apo-IscR and low amounts of [2Fe-2S]-IscR within the cell. This could explain the observed partial repression and plumbagin-induced expression of target genes. The mutation of the non-conserved C111 near the ligand ligation site generated a target gene expression pattern similar to that of wild-type IscR.

The analysis of the IscR-binding motifs on the *iscR* promoter indicates that these motifs could belong to the type 1 motif class (Figure 3A). Taken together, these findings indicate that [2Fe-2S]-IscR binding at the type 1 site is responsible for the repression of *isc* operon expression. Thus, apo-IscR is likely not involved in the regulation of the *isc* operon [8].

### Characterisation of IscR Residues Involved in Iron-sulphur Cluster Ligation

Apo-IscR atypically ligates a [2Fe-2S] cluster with three cysteines and one histidine residue [13]. To address the importance of these [2Fe-2S] cluster ligands, 6xHis-tagged IscR wild-type and IscR variants carrying amino acid substitutions at residues thought to be involved in ligation (C92A, C98A, C104A, or H107A) were purified and equal amounts of the mutant proteins were then subjected to UV-visible spectroscopy as described in Methods. Both IscR-E43A and IscR-C111A had significant absorption at 415 nm similar to the wild-type protein (Figure 3B). By contrast, the IscR variant proteins (C98A, C104A, H107A, or C92A) had lower absorption at 415 nm relative to the wild-type protein indicating a reduced amount of [2Fe-2S] clusters ligated to these IscR variants (Figure 3B). The IscR-C92A had the lowest absorption at 415 nm, indicating that [2Fe-2S] clusters bind less well to this protein. These results implicate residues C92, C98, C104, and H107 in [2Fe-2S] binding to IscR and support the qRT-PCR data (Figure 2B and C) indicating that defects in [2Fe-2S] cluster ligation affect the ability of IscR to repress transcription of the *isc* operon.

To determine the effect of ROS on [2Fe-2S] cluster integrity, purified wild-type IscR was incubated with various concentrations of hydrogen peroxide (H_2_O_2_) for 10 minutes prior to UV-visible spectroscopy. The results showed decreases in IscR absorbance at 415 nm that were H_2_O_2_ concentration-dependent, suggesting that the [2Fe-2S] cluster ligated to IscR were targets for H_2_O_2_-mediated oxidation (5–50 mM) that resulted in destabilization of [Fe-S] bound to the protein. Treatment of the protein with a high concentration (0.5 M) of H_2_O_2_ led to a total loss of [2Fe-2S] clusters bound to IscR as shown in Figure 3C. These results, together with the qRT-PCR results showing the derepression of *isc* operon transcription in the presence of plumbagin (Figure 2B and C), suggest that during oxidative stress, IscR-bound [2Fe-2S] clusters are damaged by ROS resulting in a decrease in [2Fe-2S]-IscR levels leading to derepression of *isc* operon transcription.

### High-level Expression of *iscR* Impairs Anaerobic Growth

**Figure 4:**
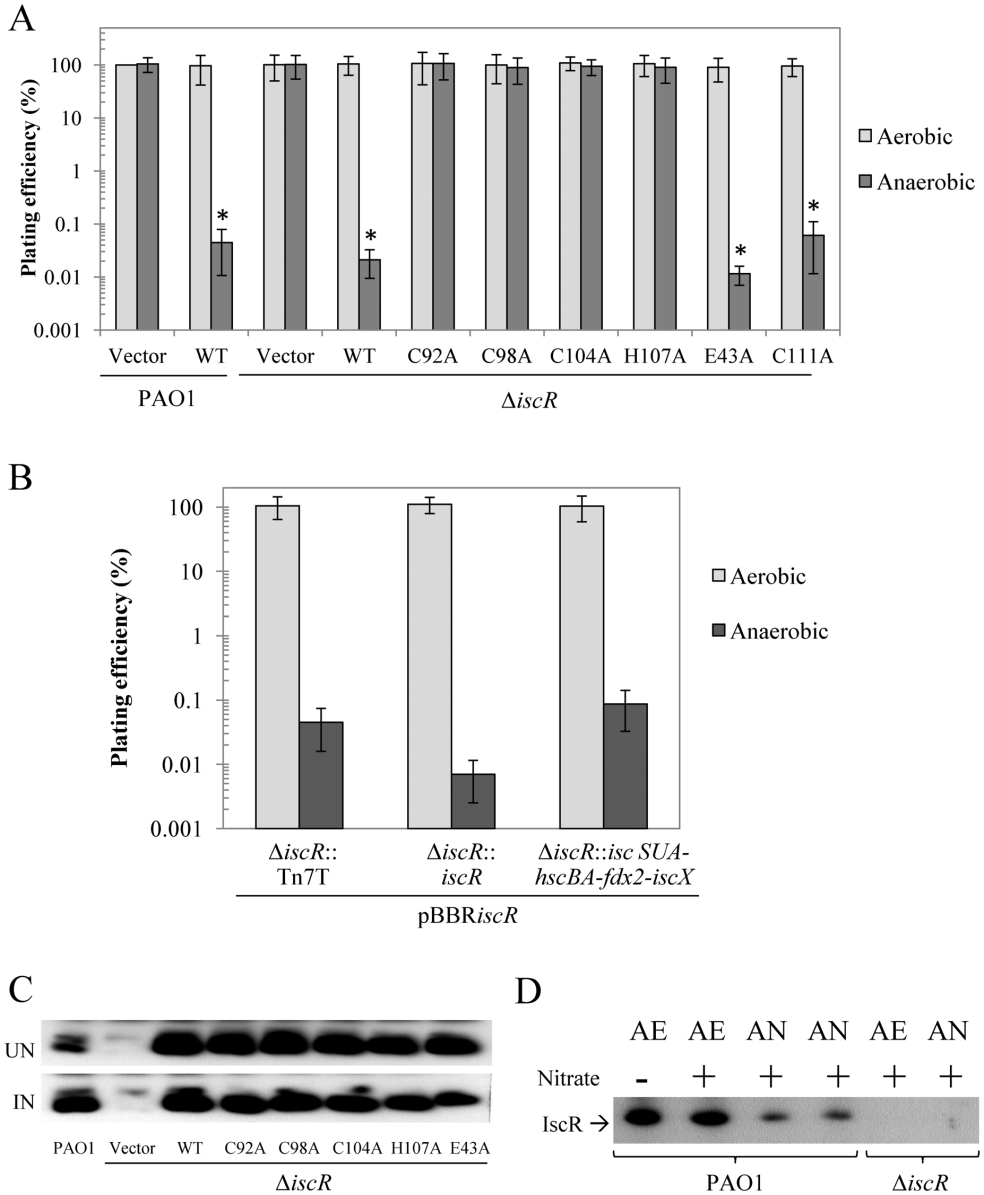
Determination of growth and IscR production in *P. aeruginosa* strains. (A) and (B) show the plating efficiencies of *P. aeruginosa* strains grown under aerobic and anaerobic conditions. (A) The PAO1 and Δ*iscR* mutants harbouring pBBR1MCS-4 (vector) along with plasmids expressing either wild-type IscR (WT), or a mutated IscR (C92A, C98A, C104A, H107A, E43A, or C111A), (B) Δ*iscR* mutants harbouring plasmids expressing wild-type IscR, as well as a chromosomal TN7 expression cassette carrying either *iscR* or *iscSUA-hscBA-fdx2-iscX*, were spread onto solid medium containing oxidants and incubated under aerobic and anaerobic conditions. Similar to the plate sensitivity assay, observations were also obtained in the absence of oxidant. The plating efficiency is defined as the number of CFU under anaerobic conditions divided by the number of CFU obtained with PAO1 harbouring an empty vector under aerobic conditions. The asterisk indicates statistically significant differences (paired *t*-test, *P*>0.05) compared with aerobic conditions. (C) and (D) Western blot analyses to determine IscR levels in *P. aeruginosa* strains. (C) Crude extracts were prepared from PAO1 and the Δ*iscR* mutant expressing wild-type and various mutated *iscR* (C92A, C98A, C104A, H107A, E43A, or C111A) grown under either uninduced (UN) conditions or induced (IN) conditions (i.e. 0.5 mM plumbagin for 30 min). (D) Crude extracts were prepared from PAO1 and Δ*iscR* mutant cultures cultivated under aerobic (AE) and anaerobic (AN) atmospheres with (+) or without (−) KNO_3_ (1%) supplementation. Electrophoresis was carried out using 15 µg protein and 12.5% SDS-PAGE.

The roles of *iscR* in aerobic and anaerobic growth were assessed. The deletion of *iscR* did not affect growth, as shown through comparable aerobic and anaerobic growth between Δ*iscR* mutant and PAO1 (Figure 4A). However, the expression of *iscR* from an expression vector strongly inhibited (5 × 10^3^-fold reduction in % plating efficiency) the anaerobic growth of both PAO1 and the Δ*iscR* mutant, while aerobic growth remained unaffected (Figure 4A). A key question is whether the levels of IscR produced from expression vectors are physiologically relevant. Western blot analysis was performed to determine the levels of wild-type IscR and mutated IscR variants (C92A, C98A, C104A, H107A, or E43A) expressed from plasmids in the Δ*iscR* mutant. The results show similar intensities of the IscR-specific bands in the *P. aeruginosa* Δ*iscR* mutant strains expressing wild-type IscR and the IscR variants (Figure 4C), indicating similar production and stability levels. The level of IscR in each strain was approximately 30-fold higher than the IscR level in PAO1 harbouring an empty vector as determined by Western blot comparison of IscR band intensity in two-fold serial dilutions of protein extracts from an IscR over-producing strain and PAO1 containing vector only (data not shown). The levels of IscR in strains harbouring expression vectors were similar to the level of IscR in wild-type PAO1 exposed to an inducer. Thus, these IscR levels are physiological relevant.

The mechanism of IscR-mediated regulation can vary with respect to both the specific target gene and the particular organism. It should be possible to use the various IscR variants to identify the physiologically relevant forms of IscR and their target genes. Phenotypic analyses of Δ*iscR* mutant strains expressing the various mutated IscR variants could provide information on the roles for the regulator in both its apo- and [Fe-S] cluster bound forms. The anaerobic growth defect phenotype was not detected in strains producing [2Fe-2S] ligation mutants (C92A, C98A, C104A, or H107A), as shown by the similar plating efficiency in anaerobic growth (Figure 4A). Moreover, the *iscR*-E43A expression in the Δ*iscR* mutant showed an anaerobic growth defect phenotype, indicating that E43 is not required for this phenotype. The Δ*iscR* mutant producing IscR-C111A, a non-conserved cysteine, also showed growth defects under anaerobic conditions similar to those producing wild-type IscR. Taken together, these results suggest that the *iscR* multi-copy inhibition of anaerobic growth likely reflects the binding of [2Fe-2S]-IscR to the type 1 binding sites of target genes. The results indicate that high levels of [2Fe-2S]-IscR adversely affect the anaerobic growth of *P. aeruginosa*. They also indicate that the ligation of [2Fe-2S] to IscR is required for inhibition of the anaerobic growth phenotype. Thus, it is unlikely that apo-IscR is responsible for this phenotype.

The inhibition of anaerobic growth by high levels of IscR could arise from repression of the *isc* operon. One of the targets for [2Fe-2S]-IscR type 1 binding is the *isc* operon, where high levels of [2Fe-2S]-IscR lead to strong repression of the *iscR* promoter that inhibit the expression of the *isc* operon, leading to the overall reduction in [Fe-S] availability in the cell. Alternatively, mis-regulation of other genes in the IscR regulon may be responsible for the phenotype. We attempted to differentiate between these possibilities by reasoning that if overly strong repression of the *isc* operon was responsible for the phenotype then unregulated expression of the genes in the *isc* operon, but not *iscR,* from a single-copy expression cassette, should alleviate the anaerobic growth inhibition phenotype. Results showed that expression of *(iscSUA-hscBA-fdx2-iscX*) from a chromosomal Tn7 expression cassette did not complement the impaired anaerobic growth of cells highly expressing *iscR* from a vector (Figure 4B). Hence, it is likely that high levels of IscR caused mis-regulation of genes in the IscR regulon other than those in the *isc* operon.

The expression levels of *iscR* under aerobic and anaerobic conditions in *P. aeruginosa* were determined using qRT-PCR. The results showed that the *iscR* expression level during anaerobic growth conditions was approximately 2.8-fold lower than the level attained during aerobic growth (Figure 2A). Furthermore, Western blots determined that the level of IscR showed a 4-fold decrease in anaerobically grown cultures compared to those grown aerobically (Figure 4D). This decrease matched the observed decrease in transcription of *iscR* under anaerobic conditions (Figure 2A). This anaerobic reduction was not simply due to the addition of nitrate since nitrate addition to aerobic cultures had no effect on the IscR level (Figure 4D). These results are consistent with the idea that the presence of oxygen reduces the proportion of [2Fe-2S]-IscR through oxidative damage to the [2Fe-2S] cluster, thereby derepressing *isc* operon transcription. This effect is amplified by increased competition for iron-sulphur cluster insertion between IscR and other damaged [Fe-S] cluster-containing proteins [21]. Under anaerobic conditions, the increased repression of the operon reflects the reduced competition for, and turnover of, [Fe-S] clusters resulting in higher [2Fe-2S]-IscR levels and increased *isc* operon repression.

### The *iscR* Mutant is Hypersensitive to Oxidants

**Figure 5:**
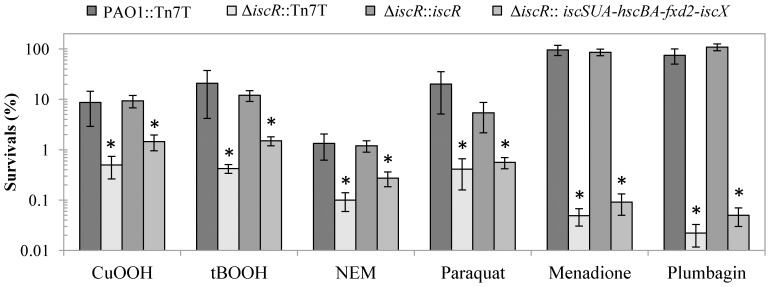
Determination of the resistance levels against stresses in *P. aeruginosa* strains. Plate sensitivity assays were performed in PAO1 and Δ*iscR* mutant transposed with Tn7T vector (PAO1::Tn7T and Δ*iscR*::Tn7T), the *iscR* complemented strain (Δ*iscR*::*iscR*), and the *isc* operon-overexpressed Δ*iscR* strain (Δ*iscR*::*iscSUA-hscBA-fdx2-iscX*) using plates containing 1.6 mM CuOOH, 1 mM tBOOH, 280 µM NEM, 250 µM paraquat, 4 mM menadione, and 1 mM plumbagin. Survival (%) is defined as the percentage of colony-forming units (CFU) on plates containing oxidant over the number of CFU on plates without oxidant. The data are presented as the means ± SD from three independent experiments. The asterisk indicates statistically significant (paired *t*-test, *P*<0.05) compared with the PAO1::Tn7T treated with the same condition.

Previous studies have shown that the *P. aeruginosa iscR* mutant is susceptible to H_2_O_2_ and paraquat. The former phenotype results from decreased catalase activity [19]. Here, we extended the investigation into phenotypic changes of the Δ*iscR* mutant to oxidative stresses. The plate sensitivity assay was used to determine the sensitivity level toward redox-cycling agents and organic hydroperoxides. The results showed that the Δ*iscR* mutant is 40- to 50-fold less resistant to organic hydroperoxides (tBOOH and CuOOH). In addition, the mutant exhibited greater than 10^3^-fold reduced resistance to redox-cycling drugs, such as plumbagin and menadione, and 50-fold reduced resistance to paraquat (Figure 5). The sensitive phenotype against both the organic hydroperoxides and redox-cycling drugs could be complemented by the expression of a single copy of *iscR* in Tn7-borne expression (Figure 5), indicating that IscR plays an important role in the oxidative stress response. In PAO1, IscR directly regulated *tpx*, a thiol peroxidase gene encoding a broad peroxide substrate thiol peroxidase [20]. It is likely that other as yet unidentified genes, directly or indirectly regulated through IscR, could be responsible for the increased sensitivity to organic hydroperoxides. In addition, a Δ*iscR* mutant containing Tn7T*iscSUA-hscBA-fdx2-iscX* did not show significant changes in oxidant resistance levels compared to the Δ*iscR* mutant suggesting mis-regulation of other genes in the IscR regulon is likely to be responsible for the increased oxidant sensitivity of the Δ*iscR* mutant. Furthermore, altered oxidant resistance in the Δ*iscR* mutant is likely a direct result of the loss of IscR, which reduces the capacity of the mutant to respond to oxidative stress.

### IscR Modulates Intracellular Iron

**Figure 6:**
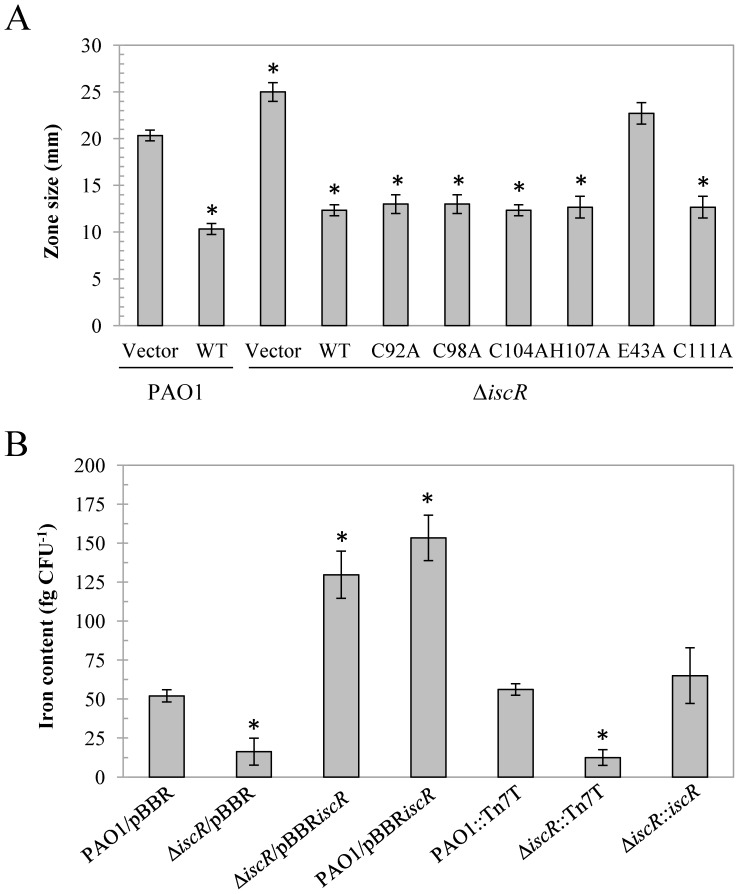
Siderophore production and intracellular iron content in *P. aeruginosa* strains. (A) Siderophore production in *P. aeruginosa* PAO1, the Δ*iscR* mutant, harbouring pBBR1MCS-4 (vector), and plasmids expressing wild-type IscR (WT) and mutated IscR (C92A, C98A, C104A, H107A, E43A, or C111A) was determined using chrome azural S (CAS) agar plates. Siderophore production is directly associated with the yellow halo zone size and the data shown are the means ± SD from three independent experiments. (B) ICP-MS was conducted as described in the Methods to measure the intracellular iron content in the indicated *P. aeruginosa* strains. The data are presented as the means ± SD from three independent experiments. The asterisk indicates statistically significant difference (*P*<0.05) compared with the PAO1 harbouring empty vector.

The availability of intracellular iron is finely balanced and controlled. The roles of *iscR* in iron homeostasis and the iron status of the mutant were investigated. Here, we have shown that the deletion of *iscR* resulted in the high-level expression of [Fe-S] biogenesis genes in the *isc* operon. Decreased intracellular labile iron in the cell could be a consequence of increased levels of [Fe-S] biosynthesis. The Δ*iscR* mutant could sense this condition as iron starvation and respond accordingly. The increased production of siderophores is one of the initial responses to low intracellular iron [24], [25]. Hence, the production of siderophores was measured in PAO1 and the Δ*iscR* mutant using a CAS assay (Figure 6A). The results showed that the Δ*iscR* mutant produced comparatively higher levels of siderophore than PAO1, based on a wider zone diameter in the CAS assay. In accordance with this observation, the over-expression of *iscR* led to the hyper-repression of siderophore expression (25 mm zone diameter for Δ*iscR* mutant compared with 10 mm zone for the mutant harbouring pBBR*iscR*) (Figure 6A). Here, we have shown that [2Fe-2S]-IscR binding to the type 1 binding site is responsible for the repression of the *isc* operon. The investigation was extended to determine whether [2Fe-2S] ligation to IscR and the E43 residue are required to complement the increased siderophore phenotype of the Δ*iscR* mutant harbouring various site-directed mutagenesis of amino acid residues involved in [2Fe-2S] ligation and the E43A mutation of *iscR*. The results illustrated that the Δ*iscR* mutants harbouring mutated *iscR* that produced IscR variants (C92A, C98A, C104A, or H107A) exhibited similar lower levels of siderophore production compared with the strain producing wild-type IscR (Figure 6A). However, the mutant expressing IscR-E43A expressed siderophores at a similar level as the Δ*iscR* mutant (Figure 6A), suggesting that the E43 residue of IscR is required for the transcription modification of genes leading to complement of the mutant increased siderophore production phenotype. Altogether, these results suggest that apo-IscR binds to the type 2 binding motif upstream of unidentified IscR target genes and activates/represses gene expression, resulting in the modulation of siderophore production. Furthermore, the results suggest that the phenotype is not a simple effect of the repression of the *isc* operon through [2Fe-2S]-IscR. Additional direct or indirect alterations in the expression of IscR-regulated genes subsequently alter siderophore production. Further experiments are currently underway to determine whether IscR directly or indirectly regulates the genes that produce this phenotype.

The total intracellular iron content in *P. aeruginosa* strains was measured using ICP-MS. Figure 6B shows that the total intracellular iron content of the Δ*iscR* mutant (12.5 fg CFU^−1^) was approximately 4-fold lower than the level observed in wild-type PAO1 (56.07 fg CFU^−1^). This reduced iron content phenotype could be restored through the expression of single copy *iscR* (65.01 fg CFU^−1^). The increased expression of *iscR* from a multi-copy vector (pBBR*iscR*) in PAO1 and the Δ*iscR* mutant increased the intracellular iron content to the levels of 129.64 fg CFU^−1^, respectively compared with PAO1 carrying the vector control (52.05 fg CFU^−1^) (Figure 6B). The Δ*iscR* mutant exhibits reduced total iron and higher siderophore levels than PAO1 (Figure 6A and B). The high-level expression of *iscR* increased the total iron content and significantly reduced siderophore production. The inactivation of *iscR* reduced the total intracellular iron content even though the production of siderophores, and presumably other iron uptake system components, was significantly increased. This suggests that changes other iron metabolism pathways, such as uptake, iron storage, or efflux, are affected through *iscR* inactivation leading to a reduction in the total intracellular iron content. Interestingly, increased siderophore production was also in a PAO1 *fur* mutant, which displayed high siderophore production but was defective in siderophore mediated iron uptake [25].

**Figure 7:**
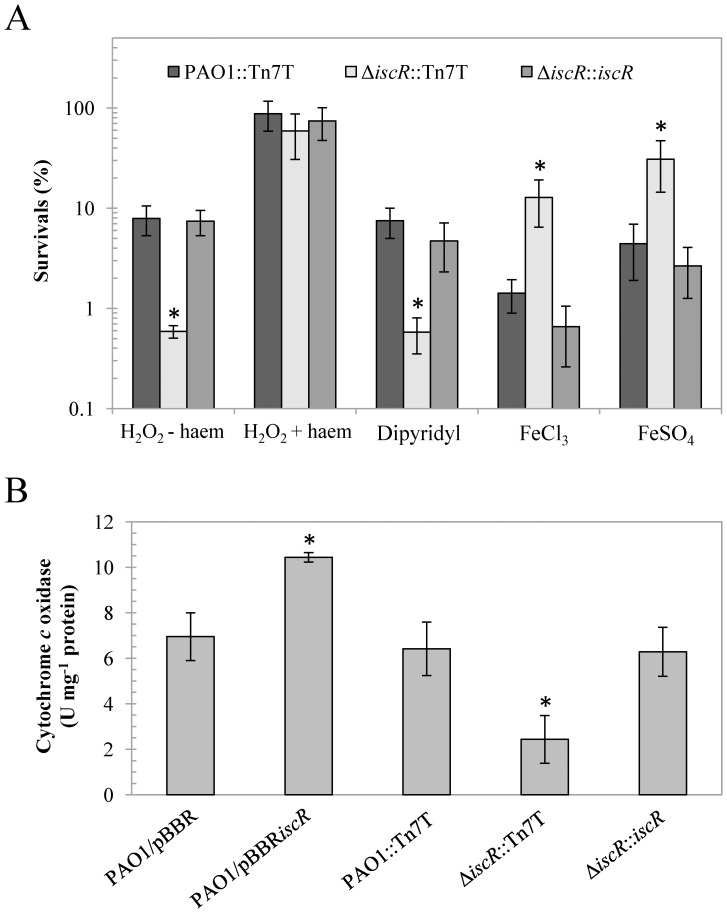
Survival under iron depletion and repletion conditions and cytochrome *c* oxidase activity in *P. aeruginosa* strains. (A) Determination of the resistance against agents affecting iron homeostasis in *P. aeruginosa* PAO1 and the Δ*iscR* mutant. Plate sensitivity assays against 0.5 mM H_2_O_2_ in the absence (−) and presence (+) of 100-µM haem, iron depletion (dipyridyl, 1.2 mM), and iron repletion (5 mM FeCl_3_ and 4 mM FeSO_4_) were performed. The asterisk indicates statistically significant (*P*<0.05) compared with the PAO1::Tn7T treated with the same condition. (B) Cytochrome *c* oxidase activity was measured in the indicated *P. aeruginosa* strains as described in the Methods. The data are presented as the means ± SD from three independent experiments. The asterisk indicates statistically significant (*P*<0.05) compared with the PAO1 harbouring empty vector.

The ability to cope with either iron deprivation or iron excess could reflect the intracellular iron status of the cells. The resistance levels against iron repletion and depletion conditions in the Δ*iscR* mutant were determined. The Δ*iscR* mutant showed decreased resistance (approximately 40-fold) to an intracellular iron chelator dipyridyl (1.2 mM), but increased resistance to the addition of both FeCl_3_ (5.5 mM) and FeSO_4_ (4 mM) (12-fold and 7-fold, respectively) relative to PAO1 (Figure 7A). Both phenotypes in Δ*iscR* mutant were fully complemented through the expression of *iscR* from a Tn7T vector. These results indicate that the Δ*iscR* mutant copes less well with iron deprivation stress, such as dipyridyl treatment than PAO1 and vice versa with excess iron conditions. These observations support the low intracellular iron conditions in Δ*iscR* mutant. The inactivation of *P. aeruginosa iscR* lowers KatA catalase activity at a posttranslational level [19]. Thus, the Δ*iscR* mutant is sensitive to H_2_O_2_ treatment [18]. KatA is a haem-containing enzyme that detoxifies H_2_O_2_ to water and oxygen and is crucial for peroxide resistance [26]. Since Δ*iscR* mutant exhibits reduced intracellular labile iron, the depletion of haem could be one of the factors responsible for the reduced KatA activity in the Δ*iscR* mutant. We observed that haem supplementation in the culture medium restored the H_2_O_2_-sensitive phenotype of the Δ*iscR* mutant (Figure 7A) and increased catalase activity (data not shown). However, it was possible that haem supplementation could complex iron thereby affecting the activity of other iron-containing enzymes involve in oxidative stress defense. In order to test this, the level of SodB, a non-haem iron-containing enzyme was measured using SOD activity gel assays, in the *P. aeruginosa* strains cultured in LB-medium supplemented with the divalent metal chelator, EDTA, or the iron-specific chelator, dipyridyl. In both cases, haem supplementation had no significant effect on SodB activity, indicating that haem was not acting as a source of free iron (data not shown). No significant difference in SodB activity was observed between the strains. This observation, combined with the fact that haem supplementation increases H_2_O_2_ resistance (Figure 7A), suggest that haem could be directly incorporated into haem-containing enzymes.

The observed increased H_2_O_2_ sensitivity suggests haem deficiency in the Δ*iscR* mutant. Therefore, we measured cytochrome *c* oxidase activity, another haem-containing enzyme in the electron transport chain. The results illustrated that the cytochrome *c* oxidase activity in the Δ*iscR* mutant (2.4 U mg^−1^ protein) was approximately 3-fold less than that in the wild-type PAO1 (6.4 U mg^−1^ protein), and this reduced enzyme activity could be restored through *iscR* in a complemented strain (6.3 U mg^−1^ protein) (Figure 7B). Taken together, the results indicate defects in the activity of haem-containing enzymes that could reflect overall reduced haem levels of the Δ*iscR* mutant.

Our data showed that *iscR* plays an important role in iron homeostasis. Inactivation of *iscR* resulted in the increased expression of the *isc* operon containing genes encoding enzymes for [Fe-S] biogenesis. This, in turn diverts the pool of labile iron towards [Fe-S] biosynthesis. Moreover, the amount of total intracellular iron was also reduced in the Δ*iscR* mutant. Other parameters of iron deprivation conditions, such as increased siderophores output, increased sensitivity to iron depletion (dipyridyl treatment), increased resistance to iron excess conditions (addition of either FeCl_3_ or FeSO_4_) and reduced haem availability as shown by reduction in haem containing enzymes levels, were observed in the Δ*iscR* mutant. The observed low total iron content in the Δ*iscR* mutant could not be correct by increased siderophore production. These results support important roles of IscR in iron homeostasis. In *P. aeruginosa*, IscR regulated target genes involved in iron homeostasis have not been characterised. Although, Fur is a regulator of iron homeostasis that regulates more than a hundred genes that participate in iron uptake, metabolism, and efflux [27]. *fur* is a possible target for IscR regulation in PAO1. Preliminary data suggest that there could be interplay between these two global regulators and their effects on target genes and overall iron homeostasis is currently being investigated.

## Methods

### Bacterial Strains and Growth Conditions

All bacterial strains and plasmids used in this study are listed in Table 1. *P. aeruginosa* PAO1 and *E. coli* strains were aerobically cultivated in Luria-Bertani (LB) broth at 37°C with shaking at 180 rpm unless otherwise stated. The medium for *E. coli* growth were supplemented with 100 µg ml^−1^ ampicillin (Ap) or 15 µg ml^−1^ gentamicin (Gm) as required, whereas the medium for *P. aeruginosa* were supplemented with 200 µg ml^−1^ carbenicillin (Cb), 25 µg ml^−1^ chloramphenicol (Cm) or 30 µg ml^−1^ Gm as required. To produce synchronous growth, an overnight culture was inoculated into fresh LB medium to give an optical density at 600 nm (OD_600 nm_) of 0.1. Exponential phase cells (OD_600 nm_ of 0.6, after 3 h of growth) were used in all experiments.

**Table 1:** Bacterial strains and plasmids used in this study.

Strain or Plasmid	Relevant characteristic(s)	Source
***P. aeruginosa***		
PAO1	Wild-type strain	ATCC15692
PAO1/pBBR	PAO1 harbouring pBBR1MCS-4	This study
PAO1::Tn7T	PAO1 transposed with pUC18-mini-Tn7T-Gm-LAC	This study
Δ*iscR*	PAO1 Δ*iscR* mutant	This study
Δ*iscR*/pBBR	Δ*iscR* mutant harbouring pBBR1MCS-4	This study
Δ*iscR*::Tn7T	Δ*iscR* mutant transposed with pUC18-mini-Tn7T-Gm-LAC	This study
Δ*iscR*::*iscR*	Δ*iscR* mutant transposed with pTn7T*iscR*	This study
Δ*iscR*::*iscSUA-hscBA-fdx2-iscX*	Δ*iscR* mutant transposed with pTn7T*iscSUA-hscBA-fdx2-iscX*	This study
***E. coli***		
DH5α	φ80d *lac*ZΔM15, *recA*1, *endA*1, *gyrA*96, *thi-*1, *hsdR*17(r_k_^−^, m_k_^+^), *supE*44, *relA*1, *deoR*, Δ(*lacYMA*-*argF*)U169	Stratagene Inc.(USA)
**Plasmid**		
pUC18-mini-Tn7T-Gm-LAC	pUC18 containing mini-Tn7T::P_lac_ site, Ap^r^, Gm^r^	[29]
pTn7T*iscR*	pUC18-mini-Tn7T-Gm-LAC containing *iscR*	This study
pBBR1MCS-4	Medium-copy-number expression vector, Ap^r^	[32]
pBBR*iscR*	pBBR1MCS-4 containing *iscR*	This study
pBBR*iscR*-C92A	pBBR*iscR* with C92A mutagenesis	This study
pBBR*iscR*-C98A	pBBR*iscR* with C98A mutagenesis	This study
pBBR*iscR*-C104A	pBBR*iscR* with C104A mutagenesis	This study
pBBR*iscR*-H107A	pBBR*iscR* with H107A mutagenesis	This study
pBBR*iscR*-E43A	pBBR*iscR* with E43A mutagenesis	This study
pBBR*iscR*-C111A	pBBR*iscR* with C111A mutagenesis	This study
pCM351	vector containing the *loxP*-Gm^r^-*loxP* region, Gm^r^	[30]

### Molecular Techniques

General molecular techniques including DNA and RNA preparation, DNA cloning, PCR amplification, Southern and Northern analyses and *E. coli* transformation were performed using standard protocols [28]. Transformation of plasmids into *P. aeruginosa* strains was carried out using electroporation as previously described [29]. The oligonucleotide primers used are listed in Table 2.

**Table 2:** List of primers used in this study.

Primer	Sequence (5′→3′)	Purpose
BT2781	GCCCGCACAAGCGGTGGAG	Forward primer for 16S ribosomal gene
BT2782	ACGTCATCCCCACCTTCCT	Reverse primer for 16S ribosomal gene
BT3210	CGAGGTAGATCGGCAATT	Reverse primer for full-length *iscR*
BT3577	CCTGCTGTCGGGTAACGC	Reverse primer for primer extension
BT3578	ATCATGCGCGAGGACTCC	Forward primer for *iscR* deletion
BT3579	GGATCGGCGTTGACCAGC	Reverse primer for *iscR* deletion
BT3555	CGCAATGGCATCGAGATCGA	Forward primer for *fdx2* expression
BT3556	GATAGCCGCGAATCGGGCTC	Reverse primer for *fdx2* expression
BT3584	CACCTGTGGGCCGACCTCAGT	Site-directed mutagenesis of IscR-C111A
BT3585	CTGAGGTCGGCCCACAGGTGG	Site-directed mutagenesis of IscR-C111A
BT3612	GAAGATTTCGCCGGAGTCAA	Forward primer for *iscR* promoter fragment
BT3613	GCGTTCGGAGATATCGGCCAG	Reverse primer for *iscR* expressionand *iscR* promoter fragment
EBI102	GCGACCCGCGCCCAGGGGCAG	Site-directed mutagenesis of IscR-C92A
EBI103	CTGCCCCTGGGCGCGGGTCGC	Site-directed mutagenesis of IscR-C92A
EBI120	ACCCCGAATGATCCCGATG	Forward primer for *iscR* expression
EBI121	GGAAAAGCCCATGCGTCTGA	Forward primer for full-length *iscR*
EBI142	CAGGGCGATGCCCACTCCGGC	Site-directed mutagenesis of IscR-C98A
EBI143	GCCGGAGTGGGCATCGCCCTG	Site-directed mutagenesis of IscR-C98A
EBI144	GGCGATACCGCTCTGACCCAC	Site-directed mutagenesis of IscR-C104A
EBI145	GTGGGTCAGAGCGGTATCGCC	Site-directed mutagenesis of IscR-C104A
EBI148	TCCTATCTCGCACAGCTGTTC	Site-directed mutagenesis of IscR-E43A
EBI149	GAACAGCTGTGCGAGATAGGA	Site-directed mutagenesis of IscR-E43A
EBI192	TGTCTGACCGCCCACCTGTGG	Site-directed mutagenesis of IscR-H107A
EBI193	CCACAGGTGGGCGGTCAGACA	Site-directed mutagenesis of IscR-H107A
M13F	GTAAAACGACGGCCAGT	Universal forward primer for expression vector
M13R	AAACAGCTATGACCATG	Universal reverse primer for expression vector

### Strain and Plasmid Constructions

The *iscR* deletion mutant was constructed using the homologous recombination with an unmarked Cre-*loxP* antibiotic marker system as previously described [30], [31]. An 1138-bp DNA fragment containing the *iscR* gene and the sequences flanking in both *iscR* termini was PCR amplified from PAO1 genomic DNA with the primers BT3578 and BT3579 and cloned into a pUC18 plasmid cut with *Sma*I, yielding pUC*iscR*. The *Kpn*I (blunted with T4 DNA polymerase) fragment containing a gentamicin resistance (Gm^r^) cassette flanked with *loxP* sequences from pUC18Gm (pUC18 containing *loxP*-flanked Gm^r^, which was constructed by inserting *Sac*I-*Eco*RI fragments containing *loxP*-flanked Gm^r^ from pCM351 [30] into pUC18 cut with the same enzymes) was cloned into pUC*iscR* digested with *Sma*I and *Eco*RV, generating pUCΔ*iscR*::Gm^r^. Digestion with *Sma*I and *Eco*RV deleted 114 bp of the *iscR* coding sequence. pUCΔ*iscR*::Gm^r^ was transferred into PAO1, and the putative Δ*iscR* mutants that arose from a double crossover event were selected for the Gm^r^ and Cb^s^ phenotypes. An unmarked *iscR* mutant was created using the Cre-*loxP* system to excise the Gm^r^ gene as previously described [30], and deletion of *iscR* was confirmed by Southern blot analysis. A pBBR*iscR* for ectopic expression of *iscR* was constructed by amplifying the full-length *iscR* from the PAO1 genomic DNA with primers EBI121 and BT3210. The 553-bp PCR products were cloned into medium-copy-number plasmid pBBR1MCS-4 [32] cut with *Sma*I, yielding pBBR*iscR*. A single-copy complementation was done using mini-Tn7 system [29]. The full-length *iscR* was cloned into pUC18-mini-Tn7T-Gm-LAC prior to transposing into the Δ*iscR* mutant, generating the complemented strain Δ*iscR*::*iscR*. Confirmation of transposition was carried out as previously described [29].

### Site-directed Mutagenesis of IscR

Site-directed mutagenesis was performed to convert cysteine (C92, C98, C104, C111), histidine (H107), or glutamic acid (E43) residues to alanine residues through PCR-based mutagenesis as previously described [20], [33]. To construct pBBR*iscR*C92A for the expression of IscR-C92A, two pairs of primers EBI103 - M13R and EBI102 - M13F, were used in two-step PCR using pBBR*iscR* as a template. The PCR product was digested with EcoRI and SacI prior to cloning into pBBR1MCS-4, generating pBBR*iscR-*C92A. pBBR*iscR*-C98A, pBBR*iscR*-C104A, pBBR*iscR*-H107A, pBBR*iscR*-C111A and pBBR*iscR*-E43A were constructed using the same protocol with different sets of mutagenic primers: EBI142 and BT143 for C98A, EBI144 and EBI145 for C104A, EBI192 and EBI193 for H107A, BT3584 and BT3585 for C111A, and EBI148 and EBI149 for E43A. The presence of each mutation was verified by DNA sequencing.

### Primer Extension

Primer extension experiments were performed using 10 µg of total RNA, ^32^P-labelled primer BT3577, 200 U Superscript II RNaseH minus M-MLV reverse transcriptase (Life Technologies, USA) and incubated at 42°C for 60 min. The primer extension products were analysed on a sequencing gel (8% polyacrylamide-7 M urea) next to DNA ladders generated using PCR sequencing kit using BT3577 and a 294-bp *iscR* promoter fragment amplified with primers BT3612 and BT3613 as template.

### qRT- PCR

Real-time reverse transcription PCR (qRT-PCR) was performed as previously described [31]. qPCR was performed using 10 ng of cDNA and primer pairs specific for *iscR* (EBI120 and BT3613), *fdx2* (BT3555 and BT3556) and the 16S rRNA gene (BT2781 and BT2782) for 40 cycles of denaturation at 95°C for 10 s, annealing at 60°C for 30 s, and extension at 60°C for 30 s. The *iscR* forward primer EBI120 corresponds to the sequence from +1 to +19 (transcribed region outside the *iscR* coding region) of the *iscR* transcript. Relative expression was shown as fold change relative to the level of wild-type PAO1 grown under uninduced conditions. The experiments were independently repeated three times and the means ± standard deviations (SD) are shown.

### Purfication of IscR and UV-visible Spectroscopy

Purification of 6xHis-tagged wild-type and mutant IscR proteins from *P. aeruginosa* was carried out using the pET-Blue2 expression system as previously described [20]. The purity of IscR protein was more than 90% as judged by a major band corresponding to the 18 kDa protein observed in SDS-PAGE. The UV-visible spectroscopy analysis of various mutated IscR variants were carried out using Shimadzu UV-1800 spectrophotometer. Equal amount of proteins (10 µM) was prepared in 50 mM phosphate buffer containing 300 mM NaCl, pH 7.0. BSA (10 µM) was used as a control. Spectroscopy analysis was immediately done after protein purification step to avoid the oxidation of the iron-sulphur cluster of the IscR proteins.

### Preparation of Polyclonal Anti-IscR Antibody

Polyclonal anti-IscR antibody was prepared by immunizing a rabbit with purified 6xHis-tagged *P. aeruginosa* IscR (conducted by the Biomedical Technology Research Unit, Faculty of Associated Medical Sciences, Chiang Mai University, Thailand).

### Western Blot Analysis

Bacterial cell lysates prepared from exponential phase cultures were separated using 12.5% SDS-PAGE and transferred to a polyvinyl difluoride (PVDF) membrane (Bio-Rad). The blotted membrane was blocked with skim milk in Tris-Buffered Saline Tween-20 (TBST) before being probed with a 1∶25,000 dilution of rabbit anti-IscR antibody as the primary antibody. Goat anti-rabbit immunoglobulin G, pAB (horseradish peroxidase [HRP] conjugated) (Enzo Life Sciences) was used as the secondary antibody. Antigen-antibody complexes were detected using chemiluminescent HRP substrate (GE Healthcare, Germany) and Hyperfilm (GE healthcare Life Sciences). Band intensity was measured using an Imagescanner (GE healthcare Life Sciences) and ImageQuant v. 1.2 software (Molecular dynamics).

### Inductively Coupled Plasma Mass Spectrometry (ICP-MS)

One milliliter of the exponential phase culture (adjusted to OD_600 nm_ of 1) was washed and resuspended in 50 mM potassium phosphate buffer solution (PBS). The cells were treated 3 times with 100 µM ethylenediamine-N, N′-diacetic acid (EDDA) in PBS prior to treatment with 300 µl of 60% ultrapure nitric acid and heated at 50°C for 18 h. The mixture was adjusted to a total volume of 2.500 ml with autoclaved Milli-Q water (ICP grade). The iron content was measured using ICP-MS. The colony forming units for each strain were determined using the viable count method and expressed as fg iron CFU^−1^.

### Plate Sensitivity Assay

A plate sensitivity assay was performed to determine the oxidant resistance level as previously described [20], [31]. Briefly, exponential phase cells were adjusted to OD_600 nm_ of 0.1 before making 10-fold serial dilutions. Then, 10 µl of each dilution was spotted onto LB agar plates containing appropriate concentrations of testing reagents. The plates were incubated at 37°C for overnight before the surviving colonies were scored. The resistance level against an oxidant was expressed as the % survival, defined as the percentage of the CFU on plates containing oxidant divided by the CFU on plates without oxidant. For anaerobic conditions, the cultured plates were incubated in an anaerobic jar containing an anaerobic gas pack (AnaeroGEN™, Oxoid, UK) for 48 h.

### CAS Assay for Siderophore Production

Siderophore production was determined using a Chrome azural S (CAS) agar diffusion assay as previously described [34]. Siderophore production is detected by the presence of a yellow halo zone around bacterial spot.

### Cytochrome *c* Oxidase Assay

Cytochrome *c* oxidase activity was spectrophotometrically measured as previously described [35]. One unit of cytochrome *c* oxidase activity is defined as the amount of enzyme required to oxidize 1 µmol ferrocytochrome *c* min^−1^ at 25°C, pH 7.0.
